# Molecular Mechanisms of Plant Abiotic Stress Tolerance: 2nd Edition

**DOI:** 10.3390/ijms27209069

**Published:** 2026-10-13

**Authors:** Michael Moustakas

**Affiliations:** Department of Botany, Aristotle University of Thessaloniki, 54124 Thessaloniki, Greece; moustak@bio.auth.gr

## 1. Introduction

Global climate change threatens to lessen future crop yields, making the molecular decoding of plant abiotic stress tolerance fundamental for sustainable agriculture [1]. To improve plant resilience to abiotic stress, a comprehensive understanding of plant stress and tolerance mechanisms is required [2,3,4]. Plants have established complex molecular networks to identify, transduce, and respond to abiotic stressors throughout a variety of adjusting paths involving signaling molecules, hormones, ion transporters, transcription factors, and epigenetic modifications [5,6,7,8,9]. Plant responses to abiotic stresses are determined by a complex interaction of aspects, involving the duration, intensity, and frequency of stress experience and the synergistic or antagonistic interactions among the diverse stressors; they also depend upon the plant species itself, the organ or tissue affected, the plant’s developmental stage, and the plant’s genetic background [1,10,11]. Once the severity of stress surpasses the plant’s adaptive capability, permanent cellular damage may appear, eventually leading to plant death [1,10,11].

## 2. Types of Abiotic Stresses and Their Impact on Plants

The most common abiotic stresses are drought and salt stress, waterlogging stress, extreme (high–low) temperatures, heavy metal pollution, nutrient deficiency, high–low light intensity, ozone pollution and mechanical wounding [2,12,13,14,15,16,17,18,19,20,21,22,23]. In response to the different abiotic stresses, plants sense stress-associated signals are able to activate complex signaling networks that coordinate the transfer of information both intracellularly and across tissues [10,11]. These signaling cascades initiate extensive transcriptional reprogramming, thereby orchestrating metabolic, physiological, and developmental responses essential for stress acclimation and adaptation [10,11,12]. The latest research has highlighted that these processes allow them to survive adverse conditions like drought, salinity, extreme temperatures, etc. [2]. Consequently, abiotic stresses exert profound effects on plant growth, physiology, and defense mechanisms, ultimately determining plant resilience, survival, and productivity under adverse conditions [12,13].

Drought and salt stress severely limit plant growth and productivity by causing severe water deficits and oxidative damage, while salt stress increases the levels of toxic ions like sodium in the plant, creating a K^+^/Na^+^ imbalance that affects many enzymatic reactions essential for plant metabolism [24,25]. Plants under water deficit conditions react by closing their stomata to preserve water, which concurrently decreases CO_2_ uptake and halts photosynthesis and plant growth, decreasing plant productivity [26,27,28]. Additionally, water deficit harms plant cell division, elongation and differentiation, eventually decreasing plant yield [27,29,30].

Drought and salt stress, and almost all environmental stresses, trigger an imbalance in reactive oxygen species (ROS) creation that induces oxidative stress, leading to cellular damage through lipid peroxidation and protein degradation; in cases in which the antioxidant mechanisms are not efficient, cell death follows [11,12,31,32,33,34,35,36,37]. ROS in plants act as critical dual role molecules serving not only as toxic cellular by-products but also as regulatory signals that link developmental control with abiotic stress responses, pathogen defense, and stomatal responses [33,35,38,39,40]. To mitigate the detrimental effects of oxidative stress, plants employ highly coordinated adaptive mechanisms involving extensive reprogramming of metabolic, molecular, and physiological processes that regulate acclimation, growth, and development by signaling networks that perceive and integrate stress-related cues and subsequently coordinate appropriate cellular and whole-plant responses [10,11,35]. A diverse array of signaling molecules and pathways mediates this complex regulatory network, enabling plants to sense changes in their environment and transmit stress signals to maintain cellular homeostasis and optimize growth and survival under water-limited conditions [10,11,35,37,41].

Waterlogging or flooding can affect large areas of agricultural land, preventing gas exchange between the topsoil and the atmosphere and consequently causing severe metabolic, physiological and structural changes in plants, leading to losses of yield of economically significant crops [42,43,44,45,46]. The hypoxic conditions in the rhizosphere of flooding areas mean that plants shift from an aerobic respiration path to an anaerobic fermentation approach, constraining the plant’s ability to absorb water and nutrients and negatively affecting the plant’s growth [44,47]. The ability of plants to acclimate to anoxic conditions is related to the formation of aerenchyma and the development of lateral roots [42].

Extreme (high and low) temperatures disturb fundamental plant processes like photosynthesis, growth, and reproduction [48,49]. High temperatures lead to plants undergoing stomatal closure in order to prevent water loss, which stops CO_2_ entry and causes declines in crop yield [48,50]. The key photosynthetic enzyme Rubisco activase is severely affected by heat stress [49]. Plants respond to acute heat stress through the accelerated transcription and translation of heat shock proteins (HSPs) [11]. The capability of a specific genotype to tolerate heat stress depends not only on the duration and the severity of the stress but also on the developmental stage of the plant [11].

Cold stress slows down cell biochemical reactions, like respiration and nutrient assimilation, and causes membrane rigidification and electrolyte leakage, which in turn restrict the movement of nutrients and water into and out of the cells, arrest growth, and reduce yield [51,52]. Plants respond to cold stress through complex physiological and molecular adjustments that regulate gene expression and protect membrane fluidity and structural integrity [53,54]. Cold stress can be classified into chilling injuries (0–15 °C) and freezing injuries (<0 °C) [55,56]. Chilling causes the membrane to become rigid, destabilizes protein complexes, and impairs photosynthesis. Freezing, on the other hand, promotes ice formation in the apoplast of plant tissues, destroys cell membranes, and leads to cell death [56,57,58]. In freezing temperature environments, plants adapt to cold stress through the synthesis of antifreeze proteins that can protect the plant’s cells from the damage of ice crystal formation within these cells [54,59].

Soils polluted with heavy metals, like cadmium (Cd), lead (Pb), zinc (Zn), chromium (Cr), arsenic (As), and mercury (Hg), have become common due to an increase in mining activities, industrial release, the increased use of fertilizers and pesticides in agriculture, and sewage sludge and municipal waste disposal [60,61,62]. Plants growing in heavy metal-polluted soils show a reduction in growth, performance, and yield [62,63]. Heavy metal phytotoxicity disrupts fundamental physiological functions, like photosynthesis and cellular homeostasis, interferes with nutrient uptake and translocation, and generates oxidative stress [61,62,64]. Heavy metal-tolerant plant species develop complicated molecular mechanisms, involving the synthesis of metal-chelating molecules, the activation of antioxidant systems, the upregulation of metal transporters, and the modulation of stress-responsive genes and transcription factors [16,64,65].

Tropospheric (ground-level) ozone pollution decreases our food supply by harming plant tissues, diminishing photosynthesis, and arresting the growth of major essential crops like maize, wheat, and soybean [18,66]. Ozone entering the plant cells may react with ethylene and other alkenes to form ROS, which damage the membrane lipids and alter ion transport, increasing membrane permeability [11]. Ozone also inhibits H^+^-pump activity and increases Ca^2+^ uptake [11].

Wounding is an environmental stress mediated by the wind, herbivore feeding, heavy rain, or hail [67,68], but is also facilitated during automatic transplanting procedures [69]. Mechanical wounding, which is a usual stress factor, stimulates various endogenous signaling molecules, and can lead to a systemic defense response [70,71].

Light is a critical environmental factor affecting plant growth and development and its intensity can vary considerably in nature, both on single leaves and plant canopies, due to seasonal changes and weather conditions [72,73,74]. The intensity and quality of light regulate photosynthesis, metabolic processes and growth patterns [75,76,77,78]. Differential photosynthetic performance from leaves of the same plant has been frequently reported for distinctive plant species [79,80]. The response of photosynthesis to changing light intensity and quality is influenced by the leaf position in the canopy, leaf age, structural differences, and plant genotype [73,80,81,82,83].

Although insufficient light availability can restrict photosynthetic performance and limit plant development, excessive light intensity may result in photoinhibition and light-induced ROS generation and oxidative damage [80,84]. However, excess light intensity that can harm the photosynthetic apparatus can be dissipated as harmless heat through the protective mechanism of non-photochemical quenching (NPQ) [33,35,85,86]. Leaf age influences sensitivity to light stress, with both young and older senescing leaves being reported to exhibit greater susceptibility [80,87]. Low-light intensity can adversely affect plant growth and development while also compromising nutritional quality [88]. Moreover, low light often results in leaf chlorosis, decreased photosynthetic activity and excessive stem elongation [89,90]. Consequently, inadequate light availability represents an important environmental constraint on crop productivity and quality.

Nutrient deficiency in plants appears when there is an absence of essential chemical elements that (a) are required to complete a plant’s life cycle, and (b) are components of an essential plant metabolite or constituent [91]. Nutrient deficiency leads to slow development, discoloration, and reduced yields [91,92]. Nutrient availability is influenced by issues like soil pH, microbial activity, moisture and temperature, but also by the plant species [92].

## 3. Molecular Mechanisms Underlying Abiotic Stress Tolerance

The articles of this Special Issue form a continuation of the articles published in the previous Special Issue [1], which also dealt with the different molecular mechanisms of plant abiotic stress tolerance.

Under different abiotic stresses like extreme temperatures, salinity, and drought, which greatly alter plant transcriptomes, the alternative splicing acts as a fast and flexible regulatory layer, emphasizing its effectiveness as a distinct mechanism of plant adaptation to abiotic stress [93]. Alternative splicing is an important post-transcriptional regulatory mechanism that increases transcriptomic and proteomic diversity by generating multiple mRNA isoforms from a single gene [93]. Alternative splicing is common within plant species but differs with developmental stage, genetic background, and environmental circumstances [94,95]. Fatima and Yoo [93], focusing on three economically important polyploid crops—*Brassica napus*, *Gossypium hirsutum*, and *Triticum aestivum*—revealed that polyploid plants, since they possess multiple sub-genomes which interact and create expanded alternative splicing complexity compared to their diploid ancestors, display enhanced phenotypic plasticity and adaptation under abiotic stresses [93]. Duplicated genes from polyploidization display differential isoforms in response to diverse environments [96]. Heat stress, for example, generally increases total isoforms and alternative splicing events, whereas cold stress reduces total isoforms per gene [96].

### 3.1. Molecular Mechanisms Underlying Osmotic Stress Tolerance

Valencia-Lozano et al. [97] performed a transcriptome analysis with highly competent somatic embryogenic lines to understand the molecular mechanisms underlying osmotic stress tolerance. By silencing the endogenous trehalase gene via an antisense approach (RD29-antTAS) in *Coffea arabica* L. cv. Typica, the somatic embryos generated marker-free, osmotic stress-tolerant lines with enhanced resilience to osmotic stress [97]. From the upregulated genes, a protein–protein interaction network (PPI) analysis was performed, which revealed the presence of the following 10 modules: the cell cycle, chromatin remodeling, somatic embryogenesis, oxidative stress, generic transcription pathway, carbon metabolism, phenylpropanoid biosynthesis, trehalose biosynthesis, proline biosynthesis, and glycerolipid metabolism [97].

Sun et al. [98] advanced our knowledge of alkaline tolerance in bread wheat and provided candidate targets for molecular breeding of saline- and alkaline-tolerant crops. Conducting an integrated genome-wide association study and transcriptome analysis identified nine significant quantitative trait loci (QTLs) and seven high-confidence candidate genes controlling alkaline stress tolerance in bread wheat (*Triticum aestivum* L.) roots [98]. The wheat tolerant genotype (Dingxi 38) maintained better root elongation, superior root structural integrity, and enhanced ROS scavenging ability compared to the sensitive line (TDP.D-27) [98]. The nine distinct alkaline-tolerance QTLs were located on chromosomes 1A, 3B, 3D, 4A, and 5B mainly mediating alkaline stress tolerance adaptation through ion homeostasis, oxidative defense, and cell wall remodeling in the bread wheat root system [98].

A whole-transcriptome RNA sequencing study [99] identified and mapped key messenger RNAs (mRNAs), microRNAs (miRNAs), long non-coding RNAs (lncRNAs), and circular RNAs (circRNAs) in sweet potato (*Ipomoea batatas* L.) adventitious roots under salt stress. Most of the 3175 differentially expressed mRNAs and 458 miRNAs were involved in key metabolic and stress-response pathways, including starch and sucrose metabolism, phenylpropanoid biosynthesis, and the MAPK signaling pathway [99]. Key miRNAs were found to act as central hubs in this network, pointing to their critical regulatory roles in how sweet potatoes tolerate salt stress [99].

The response mechanism of *Lilium pumilum* to saline–alkali stress was investigated by cloning the LpGDSL (GDSL lipase, Gly-Asp-Ser-Leu) gene [100]. The expression of LpGDSL gene in *Lilium pumilum* reached its highest level 12 h after exposure to salt and alkali stress; by increasing lignin to make plant cell walls thicker, by scavenging ROS creation to prevent cellular damage, and by maintaining chemical balance, it facilitated the plant’s survival in high salt and alkali conditions [100].

### 3.2. Molecular Mechanisms Underlying Drought Stress Tolerance

Improving wheat yield performance under water-limited conditions was examined in a study using QTL mapping in a population of 90 doubled haploid lines derived from the cross Chinese Spring × SQ1 [101]. The researchers identified 40 QTLs for agronomic traits (biomass, grain yield, thousand grain weight) and 53 QTLs for soluble carbohydrates [101]. Concurrent QTLs for multiple traits linked to carbohydrate metabolism under water deficit were found across 21 chromosomal regions on chromosomes 2D, 4A, 4B, 5B, 5D, 6B, and 7A [101]. The authors concluded that lines designated as DH39, DH51, and DH57 exhibited the best total genotype selection indices, making them ideal candidates for further inclusion in drought-resistant breeding programs [101].

The ectopic expression of the barley F-box gene *HvFBX158* enhanced drought resistance in *Arabidopsis thaliana* by regulating multiple stress-response pathways, reducing cellular damage through the increased activity of antioxidant enzymes, particularly superoxide dismutase (SOD), and promoting root growth compared to wild-type plants [102]. Transcriptome analysis confirmed that *HvFBX158* acts as a broad coordinator, modulating complex networks involved in osmotic adjustment, redox balance, and plant resilience under drought stress [102].

Quercetin, a natural plant pigment that belongs to the large group of flavonoids, possesses a powerful antioxidant capacity, and plays an important role in stimulating germination, plant growth, and photosynthesis under non-stressed or abiotic stressed conditions [103,104,105]. Exogenous application of the quercetin–copper complex (Q-Cu (II)), especially at 0.05% and 0.1% concentrations, in wheat drought-stressed plants, significantly mitigated the negative effects of drought stress by improving chlorophyll content, enhancing photosynthetic performance, and stimulating defense-related antioxidant enzymes, like guaiacol peroxidase (GPOX) [105].

### 3.3. Molecular Mechanisms Underlying Nutrient Deficiency Tolerance

Plant nitrate transporters in the NPF (NRT1) family are involved in a number of physiological processes characterized by multifunctionality [106]. Proteins in the plant NPF6 subfamily form a distinct evolutionary group and function primarily as proton-coupled transporters, having a fundamental role in nitrogen-use efficiency and stress resistance, serving as promising targets for increasing the productivity of crops and their tolerance especially under salinity stress [106].

### 3.4. Molecular Mechanisms Underlying Biotic and Abiotic Stress Tolerance

A detailed study [107] evaluated nitric oxide and nitrosative modifications in maize seedlings (*Zea mays* L.) either infested with insects, exposed to drought, or under these combined (insects and drought) stresses. Nitric oxide (NO) and other reactive nitrogen species (RNS) are considered to be signaling molecules involved in the regulation of plant growth and development [107]. An aphid-resistant cultivar displayed faster and higher NO accumulation, increased mRNA nitration, and higher expression of NO metabolism genes like *nos-ip* and *nr1*, while this early adaptive response was absent in the aphid-susceptible cultivar, which exhibited instead a higher accumulation of the harmful peroxynitrite anion (ONOO−) and protein 3-nitrotyrosine [107]. Further research including transcriptomic and proteomic analyses can provide superior insights into the highly complex NO metabolism for the identification and selection of maize cultivars with increased tolerance to environmental stresses.

### 3.5. Molecular Mechanisms Underlying Cold Stress Tolerance

Anthers are sensitive to cold stress, which also decreases pollen vitality and quantity [108]. A study by Guan et al. [108] sheds light on molecular mechanisms of anther adaptation to cold stress in tomato (*Solanum lycopersicum* L.). Cold exposure of tomato plants triggered increased expression of *SlHB8* in the tapetum layer, and mutants lacking *SlHB8* show higher pollen viability, increased pollen tube germination rates, and significantly better fruit-set rates under cold stress conditions [108].

The articles in this Special Issue deal with the different molecular mechanisms of plant tolerance to abiotic stresses with the aim of contributing to a better understanding of plant tolerance mechanisms to stress factors, which can help to mitigate abiotic stress impacts on plants, increase agricultural productivity, and support food security.
